# Effects of Mild Traumatic Brain Injury on Resting State Brain Network Connectivity in Older Adults

**DOI:** 10.1007/s11682-022-00662-5

**Published:** 2022-04-08

**Authors:** Mayra Bittencourt, Harm-Jan van der Horn, Sebastián A. Balart-Sánchez, Jan-Bernard C. Marsman, Joukje van der Naalt, Natasha M. Maurits

**Affiliations:** 1 Department of Neurology, University of Groningen, University Medical Center Groningen (UMCG), Groningen, The Netherlands; 2 Department of Biomedical Sciences of Cells and Systems, University of Groningen, University Medical Center Groningen, Cognitive Neuroscience Center, Groningen, The Netherlands

**Keywords:** Brain connectivity, Aging, Post-concussive symptoms, Mild traumatic brain injury, Fusiform gyrus

## Abstract

Older age is associated with worsened outcome after mild traumatic brain injury (mTBI) and a higher risk of developing persistent post-traumatic complaints. However, the effects of mTBI sequelae on brain connectivity at older age and their association with post-traumatic complaints remain understudied.

We analyzed multi-echo resting-state functional magnetic resonance imaging data from 25 older adults with mTBI (mean age: 68 years, SD: 5 years) in the subacute phase (mean injury to scan interval: 38 days, SD: 9 days) and 20 age-matched controls. Severity of complaints (e.g. fatigue, dizziness) was assessed using self-reported questionnaires. Group independent component analysis was used to identify intrinsic connectivity networks (ICNs). The effects of group and severity of complaints on ICNs were assessed using spatial maps intensity (SMI) as a measure of within-network connectivity, and (static) functional network connectivity (FNC) as a measure of between-network connectivity.

Patients indicated a higher total severity of complaints than controls. Regarding SMI measures, we observed hyperconnectivity in left-mid temporal gyrus (cognitive-language network) and hypoconnectivity in the right-fusiform gyrus (visual-cerebellar network) that were associated with group. Additionally, we found interaction effects for SMI between severity of complaints and group in the visual(-cerebellar) domain. Regarding FNC measures, no significant effects were found.

In older adults, changes in cognitive-language and visual(-cerebellar) networks are related to mTBI. Additionally, group-dependent associations between connectivity within visual(-cerebellar) networks and severity of complaints might indicate post-injury (mal)adaptive mechanisms, which could partly explain post-traumatic complaints (such as dizziness and balance disorders) that are common in older adults during the subacute phase.

**Supplementary Information:**

The online version contains supplementary material available at 10.1007/s11682-022-00662-5.

## Effects of Mild Traumatic Brain Injury on Resting State Brain Network Connectivity in Older Adults

Mild traumatic brain injury (mTBI) is a leading public health issue worldwide (Carroll et al. 2014; Levin and Diaz-Arrastia 2015). Old and young adults are at highest risk of sustaining an mTBI (Bruns and Hauser 2003). With the world’s population increasing and ageing, the older population with mTBI will remain a growing group in the years to come (Roozenbeek et al. 2013). Self-reported cognitive, physical and affective complaints are common after mTBI and usually resolve within a few weeks. However, a subgroup experiences post-traumatic complaints (PTCs) that persist for months or years, which is often referred to as post-concussive syndrome (PCS; Levin and Diaz-Arrastia 2015). Older adults are more likely to experience slower recovery trajectories and persisting PTCs than their younger counterparts (King 2014). Nonetheless, little is known so far about the mechanisms behind the development of persisting PTCs at older age (Carroll et al. 2004; Kristman et al. 2014).

In terms of neurophysiology, TBI can cause diffuse axonal injury and focal brain damage (Johnson et al. 2013). Although growing evidence supports that damage might occur even if injury severity is mild, this cannot be detected using conventional neuroimaging techniques (Sharp et al. 2014). Functional magnetic resonance imaging (fMRI) is an advanced neuroimaging technique that allows the investigation of brain networks linked to various domains of functioning (e.g. cognition, vision). Particularly, resting state fMRI (rs-fMRI) is a suitable modality to study how aging and several neuropathologies, such as TBI, affect the brain. In healthy participants, consistent patterns for resting state networks connectivity have been found (see Smith et al. (2013) and van den Heuvel and Hulshoff Pol (2010) for reviews). Thus, investigating altered patterns of brain network connectivity can provide insight on how aging and mTBI might affect the brain and the development of complaints after brain injury. Previous studies have investigated brain network connectivity alterations at older age (vs. younger age) (Geerligs et al. 2015), during healthy aging (Allen et al. 2011), after mTBI (see Rosenthal et al. (2018) and Sharp et al., (2014) for reviews) and the interaction between mTBI and aging during adulthood (Bittencourt-Villalpando et al. 2021). With ageing, ICNs throughout the brain undergo profound changes in their connectivity patterns. This reorganization process results in weaker long-distance between-network connectivity and stronger short-distance between-network connectivity (Damoiseaux 2017). Besides, ICNs that are involved in high-order cognitive processes become less specialized and less differentiated from each other (Geerligs et al. 2015). This phenomenon is thought to be part of (attempted) compensatory mechanisms for sustaining cognitive performance by recruiting additional neighboring neural resources, which may be successful or not (Grady 2012).

By contrast, the effects of mTBI on brain connectivity are less clear. Abnormalities later than one month post-injury are thought to be associated with (the development of) PCS and/or neuropsychiatric conditions (Rosenthal et al. 2018; Stevens et al. 2012; van der Horn et al. 2016). Furthermore, aging is an important confounder in mTBI studies. Notwithstanding the effects of aging in brain connectivity are stronger than those of mTBI, most studies do not address age in their analysis beyond age-matching patient and control groups (Bittencourt-Villalpando et al., 2021). To date, the associations between the development of PTCs and alterations in brain connectivity are unclear and little is known about the effects of mTBI on brain connectivity in older adults.

The current study is the first to investigate brain network connectivity specifically in older adults with mTBI (OA-mTBI; age ≥ 60 years old). Here, we analyzed within- and between-network connectivity of intrinsic connectivity networks (ICNs), following a similar multivariate approach as previously used by our research group to investigate the effects of ageing and mTBI in a different adult population (age range: 18–65 years, Bittencourt-Villalpando et al., 2021). It is known that ageing affects both between- and within-network connectivity (Damoiseaux 2017; Geerligs et al. 2015). Here, we hypothesize that mTBI acts as a stressor on brain functioning and that, at older age, fewer resources are available to cope with the injury. In light of our recent work (Bittencourt-Villalpando et al. 2021), we expect that the most prominent age- and/or mTBI-related effects on brain network connectivity would involve cognitive, default mode, and/or cerebellar networks, and that perturbations of these networks might underlie persistent complaints in older adults with mTBI. Therefore, an additional research aim was to explore possible associations between severity of (post-traumatic) complaints with altered rs-fMRI brain network connectivity.

## Methods

### Participants

Data from OA-mTBI (age > 60 years) and age-matched (older adults) healthy controls (OA-HC) were obtained as part of a larger prospective follow-up study (ReCONNECT-study). Patients were included at the University Medical Center Groningen (UMCG), the Netherlands (a level 1 trauma center) between November 2018 and November 2020. The diagnosis of mTBI was based on the following criteria: attending the hospital with an mTBI defined by a Glasgow Coma Scale score of 13–15, loss of consciousness ≤ 30 min and/or post-traumatic amnesia of ≤ 24 h (Kay et al. 1993). Inclusion criteria were comprehension of the Dutch language and age 60 years or older. Healthy controls were recruited via social contacts and advertisements. For both groups, exclusion criteria were: a history of drug or alcohol abuse, a major psychiatric or neurologic disorder (e.g. schizophrenia, bipolar disorder, major depressive disorder, and dementia) as previously diagnosed or identified by the attending or ward physician and/or a previous hospital admission due to a TBI. The ReCONNECT-study was approved by the Medical Ethical Committee of the UMCG; written informed consent was obtained from all participants. All procedures were performed according to the declaration of Helsinki (2013).

### Current and Pre-Injury Severity of Complaints

The Head Injury Symptoms Checklist (HISC) is a 21-item post-traumatic questionnaire (de Koning et al. 2016), derived from the Rivermead Post-concussion Symptoms Questionnaire (King et al. 1995). The HISC was administered to all participants on the day of the fMRI scan. One of the 21 complaints, namely intolerance to alcohol, was excluded from the analysis as most patients left a remark on the questionnaire explaining that they did not consume alcohol since the injury. The HISC scores the current and the pre-injury severity level of each complaint with values ranging from 0 to 2 (never = 0, sometimes = 1, and often = 2). For OA-HCs, only current severity level of complaints score (HISC-sev) can be assessed. The HISC-sev was then calculated as the sum of the severity of all the 20 current complaints assessed (ranging from 0 to 40). For OA-mTBI, additionally, post-traumatic symptoms were defined as new complaints or complaints that were increased after mTBI (i.e. a positive result after the subtraction of pre-injury from current scores, for each complaint).

Only current severity level of complaints score (HISC-sev) was used in further statistical analysis. The HISC-sev scores were first square root (sqrt)-transformed to achieve a normal distribution (sqrt(HISC)).

### FMRI Acquisition

A Siemens MAGNETOM Prisma 3 T MRI scanner (Siemens, Erlangen, Germany) equipped with a 64-channel SENSE head coil was used for image acquisition. A high-resolution transversal T1-weighted image was made for anatomical reference (repetition time [TR] 2300 ms; echo-time [TE] 2.98 ms; flip angle [FA] 9°; field of view [FOV] 256 × 240 × 176 mm; voxel size 1 × 1 × 1.2 mm). For resting-state imaging, T2*-weighted echo planar imaging volumes were acquired with a multi-echo-EPI sequence (TR 2000 ms, TE 9.74, 22.10 and 34.46 ms; FA = 60°; FOV 256 × 256 mm; voxel size 3.5 mm-isotropic) (Feinberg et al. 2010; Moeller et al. 2010; Xu et al. 2013).

Patient rs-fMRI data were acquired at approximately five weeks after injury. All participants were instructed to close their eyes and to stay awake (duration: 10 min, 300 volumes).

### FMRI Preprocessing

First, multi-echo ICA (ME-ICA; meica.py script version 3.2; Kundu et al. 2012, 2017) was applied for denoising and image realignment to the first functional image. Afterwards, Statistical Parametric Mapping (SPM12, http://www.fil.ion.ucl.ac.uk/spm/, Ashburner & Friston, 2005) implemented in Matlab (version R2020a; MathWorks, Natick, MA) was used. The SPM preprocessing pipeline consisted of co-registration of functional images with individual participants’ T1-weighted images and normalization to the Montreal Neurological Institute template using a diffeomorphic nonlinear registration tool (DARTEL; isotropic voxels of 3 mm; smoothing 6 mm full-width-at-half-maximum Gaussian kernel). The first five volumes from each participant were excluded from the analysis for T1 equilibration.

### Group ICA

We used Group ICA fMRI toolbox (GIFT; version 4.0c; Calhoun et al. 2001) for the identification of ICNs. This approach is described in the Appendix.

### Statistical Analysis

For the set of *C* identified ICNs, we assessed two measures of functional connectivity: spatial map intensity (SMI) and (static) functional network connectivity (FNC) as measures of within- and between-network connectivity, respectively.

The design matrix included four covariates of interest: group as a categorical variable (OA-mTBI or OA-HC), severity of complaints score (i.e. HISC-sev) as a continuous variable, age as a continuous variable (because it has been identified as a strong confounder for mTBI effects in mid-adulthood (Bittencourt-Villalpando et al., 2021)) and the interaction term group × HISC-sev. Additionally, we included average framewise displacement (FD; in mm; Power et al. 2012) as a nuisance covariate to control for head motion. FD was first dichotomized into a categorical variable (high- and low-movement) using median split due to its skewed distribution.

We followed a multivariate approach as proposed by (Allen et al., 2011) using the MANCOVAN toolbox, which is implemented in GIFT. The MANCOVAN implementation tests the explained variance of each covariate for each of the functional connectivity measures using multivariate analysis of covariance and performs backward selection of the model terms. The purpose of this procedure is to select important covariates before performing the univariate tests. For more details on the MANCOVAN implementation, please refer to Allen et al., (2011).

For SMI results, in case of main or interaction effects for the covariates of interest, z-scores were averaged over all significant voxels exhibiting significant effects of the same sign (positive or negative). The results were visualized using boxplots.

We calculated the Pearson correlation coefficients (r) to measure the strength of the linear relationship between continuous variables of interest (e.g. sqrt(HISC)) and the dependent variables using the “corr” function implemented in Matlab. The results were visualized using scatter plots.

Testing for group differences was done in IBM SPSS Statistics, Version 26.0 using independent T-tests for normally distributed continuous variables and Pearson Chi-square tests for categorical variables.

All tests were corrected for multiple comparisons at an α = 0.05 significance level using false discovery rate (FDR; Genovese et al. 2002) correction.

### Post-hoc Analysis

We performed an additional post-hoc analysis to verify the association between complaints reporting (per complaint domains) and altered brain connectivity. First, complaint domains were determined according to a modularity analysis using HISC data that were obtained from a larger mTBI population (UPFRONT study; van der Naalt et al. 2017). The post-hoc analysis is detailed in the Appendix.

## Results

**Fig. 1:**
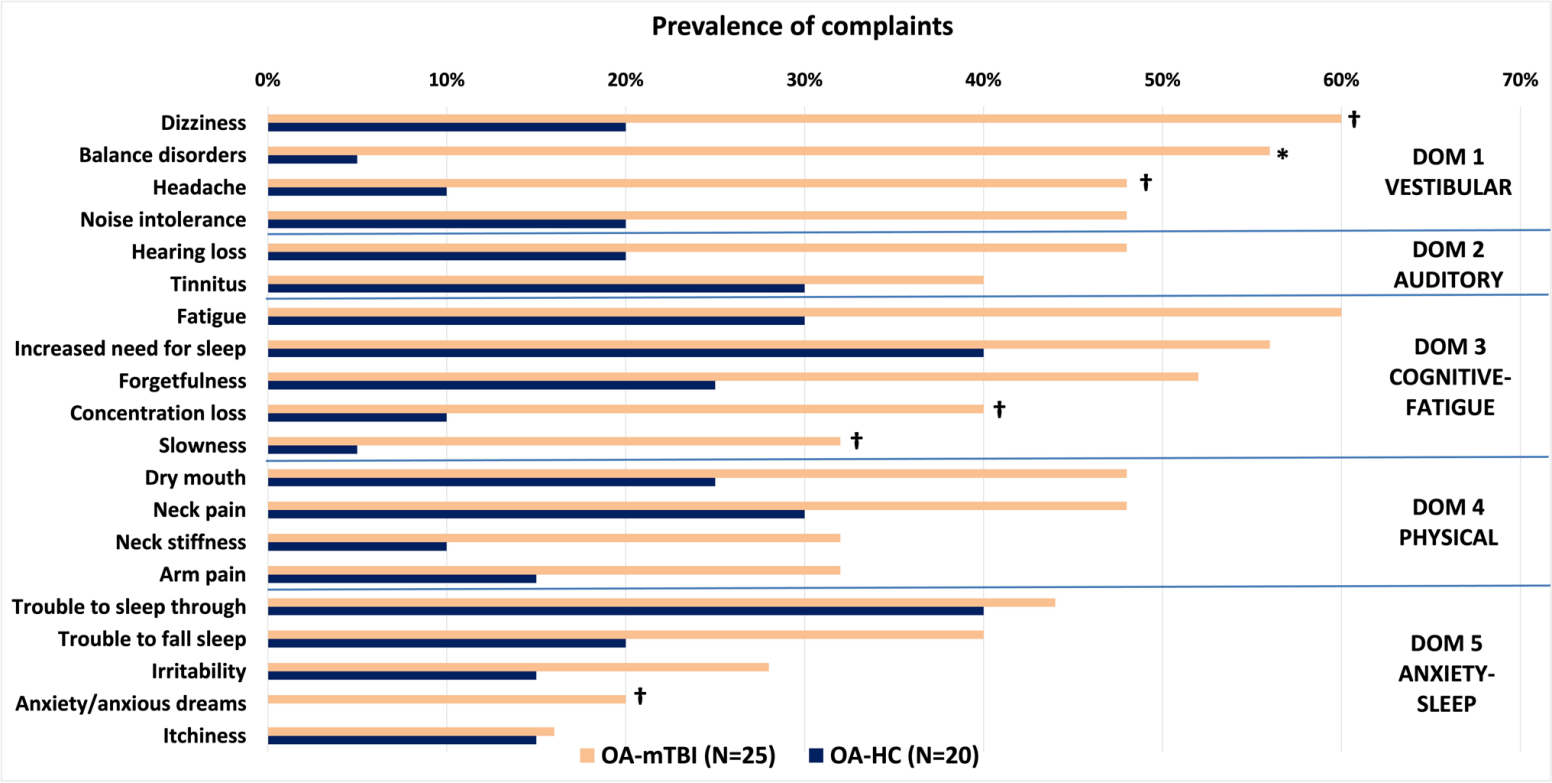
**Overview of the prevalence of complaints (%) per group** and per domain (OA-mTBI: older adults with mTBI; OA-HC: older adults-healthy controls; DOM: complaint domain). Complaints ordered by prevalence in the older adults with mTBI group from highest to lowest within each of the five identified complaint domains. Asterisks indicated significant differences between groups (Chi-square tests; *p < 0.05, FDR-corrected; †p < 0.05, uncorrected)

The summary of participant characteristics per group is presented in Table 1. We did not identify any significant differences for age, sex, nor FD between groups. Older adults with mTBI had higher (current) severity of complaints than OA-HC (p < 0.001). Additionally, pre-injury severity complaints scores reported by OA-mTBI were similar to current severity complaints scores reported by OA-HCs (p = 0.47). An overview of the prevalence of individual complaints per group (OA-mTBI or OA-HC) is presented in Fig. 1. The prevalence of post-traumatic symptoms within the OA-mTBI group can be found in the Appendix, Fig. S1.

**Table 1:** Participant characteristics per group

	**OA-HC** **(N = 20)**	**OA-mTBI** **(N = 25)**	**p-value**
**Age, mean (SD), years**	67 (5)	68 (5)	0.879^a^
**Sex, females/males**	9/11	9/16	0.540^b^
**Sqrt(HISC), mean (SD), current score**	1.9 (1.0)	3.1 (1.0)	< 0.001^a^
**Sqrt(HISC), pre-injury score**	-	2.1 (0.9)	-
**FD, median (IQR), mm**	0.17 (0.18)	0.16 (0.11)	0.529^b^
**Interval injury to scan, mean (SD), days**	N/A	38 (9)	N/A
**GCS, number of patients**			
15	N/A	13	N/A
14	N/A	8	N/A
13	N/A	4	N/A
**Injury Mechanism, number of patients**			
Traffic (bicycle/e-bike)	N/A	14	N/A
Traffic (other)	N/A	1	N/A
Falls	N/A	9	N/A
Assault	N/A	1	N/A

^a^Independent-samples t-test; ^b^Pearson Chi-square test; FD: Framewise Displacement; GCS: Glasgow Coma Score; IQR: interquartile range; OA-HC: older adults-healthy controls; OA-mTBI: older adults with mild traumatic brain injury; SD: standard deviation; sqrt(HISC): square root transformed severity of complaints score.

### Group ICA and Statistical Analysis

The group ICA resulted in *C* = 15 ICNs, which were grouped into five functional domains, one of which defined as a mixed domain (i.e. ICNs with high activation in more than one functional domain) (see Appendix, Fig. S2).

The results from the multivariate tests representing the significance of the covariates of interest in predicting SMI and FNC for the 15 identified ICNs are shown in the Appendix, Fig. S3. None of the covariates of interest were retained as predictors for FNC.

Subsequently, we identified for which ICN regions (voxels) the SMI were associated with the retained covariates of interest.

**Fig. 2:**
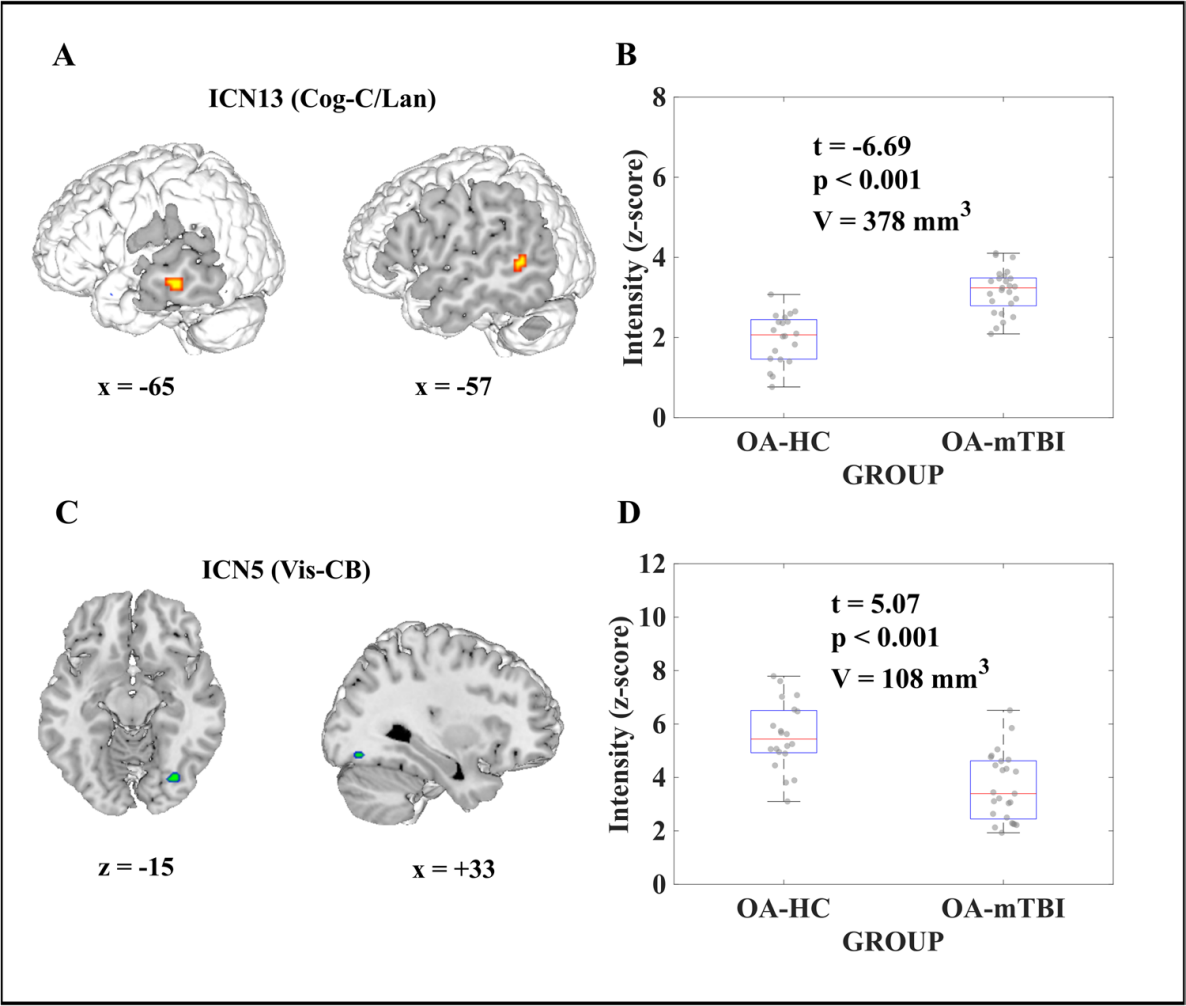
**Effects of group on spatial map intensity (SMI).** (A, C): Significant effect of group (older adults with mild traumatic brain injury; OA-mTBI vs. older adults-Healthy controls; OA-HCs) for SMI of intrinsic connectivity network (ICN) showing increased SMI for OA-mTBI in the left middle temporal gyrus on ICN13, and decreased SMI for OA-mTBI in the right posterior fusiform gyrus on ICN5 (p < 0.05, FDR-corrected) in representative slices. (B, D): Boxplots of the SMI averaged over all significant voxels across participants, per group. Cog-C/Lan: Cognitive-language domain; Vis-CB: Visual(-cerebellar) domain

The main effects of group on SMI are shown in Fig. 2. In comparison to healthy counterparts, OA-mTBI showed increased SMI in the left middle temporal gyrus (lMTG)(ICN13; Cog-C/Lan; t = -6.69, p < 0.001, V = 378mm^3^; Fig. 2A-B) and decreased SMI in the right posterior fusiform gyrus (ICN5; Vis-CB; t = 5.07, p < 0.001, V = 108mm^3^, Fig. 2C-D).

**Fig. 3:**
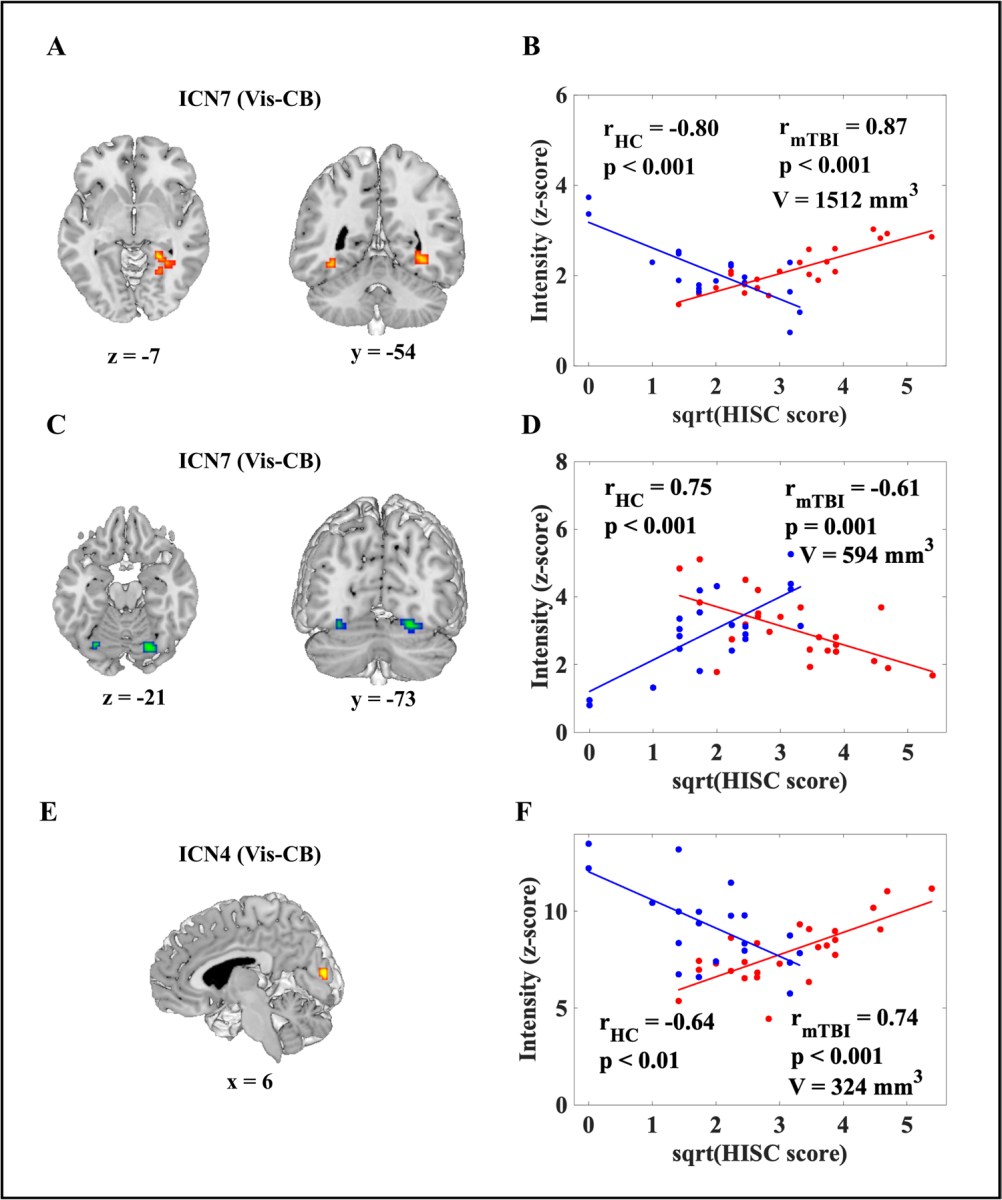
**Group × sqrt(HISC) interaction effect on spatial map intensity (SMI) of older adults with mild traumatic brain injury (OA-mTBI) and older adults HCs (OA-HCs).** (A, C, E): Voxels with significant Group × sqrt(HISC) interaction effect for SMI of intrinsic connectivity network (ICN) in the anterior fusiform and middle occipital gyri on ICN7 (A), in the cerebellum VI and Crus I bilaterally on ICN7 (C) and in the cuneus on ICN4 (E) (p < 0.05, FDR-corrected). (B, D, F): Scatterplot of sqrt(HISC) against SMI (z-score) averaged over all significant voxels across participants. Blue: OA-HCs; red: OA-mTBI; sqrt(HISC): square root transformed severity of complaints score. Vis-CB: Visual(-cerebellar) domain

Interaction effects of sqrt(HISC) × group on SMI are shown in Fig. 3. In the anterior fusiform and middle occipital gyri (ICN7; Vis-CB), SMI decreased with sqrt(HISC) in OA-HC, but increased with sqrt(HISC) in OA-mTBI (OA-HC: r = -0.80; p < 0.001; OA-mTBI: r = -0.87, p < 0.001; V = 1512mm^3^; Fig. 3A-B).

In the cerebellum VI and Crus I bilaterally (ICN7; Vis-CB), SMI increased with sqrt(HISC) in OA-HC, but decreased with sqrt(HISC) in OA-mTBI (OA-HC: r = 0.75; p < 0.001; OA-mTBI: r = -0.61; p = 0.001; V = 594mm^3^; Fig. 3C-D).

In the cuneus ICN4(Vis-CB), SMI decreased with sqrt(HISC) in OA-HC, but increased with sqrt(HISC) in OA-mTBI (OA-HC: r = -0.64; p < 0.01; OA-mTBI: r = 0.74, p < 0.001; V = 324mm^3^; Fig. 3E-F).

### Post-hoc Analysis

The results of the post-hoc analysis can be found in the Appendix.

## Discussion

In this study, we examined fMRI-ICNs in a sample of OA-mTBI and age-matched OA-HCs. We employed an ICA-based multivariate approach to identify the effects of mTBI on brain network connectivity, severity of complaints and their interaction at older age. We identified main effects of mTBI on network connectivity within visual(-cerebellar) and cognitive-language networks. Additionally, we identified interaction effects between severity of complaints and group on network connectivity within visual(-cerebellar) networks, suggesting that these regions are the most affected in older adults that have sustained an mTBI. No effects on between-network connectivity were found. Our findings are discussed below.

We found increased within-network connectivity for OA-mTBI in comparison to OA-HCs in clusters located in the left middle temporal gyrus (lMTG) of the cognitive-language ICN. The lMTG is involved in language processing, which requires a complex integration of sensory inputs (e.g. visual and auditory) (Fridriksson et al. 2016; Hickok and Poeppel 2007). Hyperconnectivity is a common finding after TBI, but its adaptive or maladaptive nature remains unclear (Hillary and Grafman 2017; Morelli et al. 2021). Previous mTBI studies using task-fMRI in younger cohorts found that hyperconnectivity in functional networks involved in attentional and visual processes might be associated with increased subjective effort and task-related fatigue (Prak et al. 2021). In our cohort, fatigue (together with dizziness) was the most prevalent complaint after mTBI, being present in 60% of the OA-mTBI (see Fig. 1). Additionally, in our post-hoc analysis we found that connectivity within the lMTG increased with severity of complaints in both cognitive-fatigue and vestibular domains, which includes noise intolerance complaints (see Appendix, Table S1). Perhaps increased awareness of external sensory stimuli could partly explain increased fatigue that is commonly experienced in elderly after mTBI.

Additionally, we found decreased within-network connectivity for OA-mTBI in comparison to OA-HCs in a cluster located posteriorly in the right fusiform gyrus (rFG) of a visual-cerebellar ICN. The rFG, particularly, is known for its role in visual pattern recognition (Grill-Spector et al. 2006), including facial recognition (Kanwisher et al. 1997). Our findings are consistent with previous studies that identified hypoactivity within visual networks of patients in the (sub)acute phase after mTBI (Amir et al. 2021; Stevens et al. 2012). Moreover, it has been suggested that the subacute phase after mTBI is marked by hypoactivation in several (non-DMN) areas across the brain, which normalizes in those patients who show good recovery (Bharath et al. 2015; Rosenthal et al. 2018). We encourage future research to investigate the longitudinal effects of mTBI on brain connectivity in the visual cortex.

In this study, we found interactions between the severity of complaints and group (OA-HC and OA-mTBI) for within-network connectivity. In clusters anteriorly located in the fusiform gyri (FG), in the middle occipital gyri and in the cuneus of two visual(-cerebellar) ICNs, within-network connectivity increased with severity of complaints score in OA-mTBI, but decreased with severity of complaints in OA-HCs. The anterior FG and the middle occipital gyri are involved in both semantic and visual processing whereas the cuneus is part of the primary visual cortex. Furthermore, in clusters located in the cerebellum bilaterally, within-network connectivity and severity of complaints score were negatively correlated in OA-mTBI, but positively correlated in OA-HCs. These results might reflect a difference in the association between brain functioning and (a high number of/more severe) complaints that are new or have recently increased in severity after a traumatic event (i.e. mTBI) and the association between (a low number of/less severe) complaints that people have been dealing with for a longer time (OA-HC). Interestingly, the cerebellum seems to play a key role in our findings. This might not be surprising considering the importance of this brain structure in integration of sensory, motor, cognitive and emotional information (Buckner 2013; Schmahmann 2019). It could be hypothesized that mTBI in older adults especially affects those networks that are responsible for multimodal integration. This is an interesting avenue for further research.

The question is how our findings can be related to the functional role of the cerebellum and of the visual cortex in light of previous research. Previous studies identified an association between PTCs and altered brain connectivity after mTBI (see van der Horn et al. 2016 and Biagianti et al. 2020 for reviews). A few of these studies identified connectivity within visual and/or cerebellar areas among those associated with PTCs (Nathan et al. 2015; Palacios et al. 2017; Stevens et al. 2012), although no clear pattern for their association emerged. It is known that, after mTBI, vision impairment including blurred vision and altered oculo-vestibular reflexes are common, although the etiology of these symptoms is still not well understood (Fife and Kalra 2015). In our OA-mTBI cohort, dizziness was the most prevalent self-reported symptom and complaint (see Fig. 1 and Appendix, Fig. S1) and complaints of balance disorders were (significantly) higher than in the OA-HC group, indicating that vestibular impairments might have been present during the subacute phase after trauma. It is therefore tempting to suggest a possible association of activity in the visual(-cerebellar) domain with vestibular impairments after mTBI. Perhaps the observed increasing hyperactivation in the visual cortex of OA-mTBI with severity of complaints score indicates increased effort to compensate for functional deficits, including oculo-vestibular impairments. However, as we do not have a direct association of vestibular and/or visual functioning measures with brain activity, such suggestion is speculative. Nevertheless, in our post-hoc analysis we found that, in OA-mTBI, connectivity within cerebellar areas decreased with severity of complaints in the vestibular domain, whereas connectivity within visual areas (i.e. anterior FG, middle occipital gyri and cuneus) increased with severity of complaints in the vestibular domain, among other domains (see Appendix, Table S1). Future studies are required to verify if a direct association of hypoactivation in the cerebellar area with vestibular impairments in OA-mTBI exists and elucidate if (attempted) compensation via increased activation in the visual cortex is part of this scene.

Regarding between-network connectivity (i.e. FNC), we did not find any effects for age or mTBI. In a previous study of our research group where we used a similar approach to analyze a younger mTBI population, we found that age strongly affected between-network connectivity during mid-adulthood, whereas no effects of mTBI were found (Bittencourt-Villalpando et al. 2021). Of note, patient and control groups were age-matched in both studies. We will discuss mTBI- and age- related findings on between-network connectivity in this order. First, there is still no consensus in the literature regarding the effects of mTBI on between-network connectivity due to lack of consistency and repeatability across studies. By employing a similar methodology and robustly controlling for age-related effects, both of our studies were consistent in identifying no effect of mTBI on FNC, suggesting that sustaining an mTBI does not affect between-network connectivity (at rest) regardless of age. Furthermore, in both of our studies, we opted to scan patients at approximately five weeks after the injury, to explore abnormalities in brain connectivity that might be associated with persistent symptoms instead of short-lived ones. Since the optimal interval for this purpose remains unknown, it is possible that shorter or longer injury to scan intervals would lead to different results. Second, the lack of age-related effects on brain connectivity in the present study might be explained by the smaller age range of its population (i.e. 20 years instead of 47 years) and/or by larger variability in age-related effects at older age; additionally, it concurs with the notion that changes in brain network connectivity occur at a slower pace at older age.

To the best of our knowledge, this is the first study to investigate (whole-)brain network connectivity in OA-mTBI. Our approach for denoising using multi-echo ICA resulted in all ICs identified as ICNs (as opposed to artifacts). Nevertheless, this study entails some limitations. First, in older adults, co-existing visual deterioration (that generally occurs as part of the aging process; Chou et al. 2016) could partly contribute to altered connectivity in visual networks and motor-balance control. However, objective measures of pre- and post-injury visual and motor-balance functioning were not available, which limits our analysis. Second, although the fMRI scan environment is noisy and uncomfortable, it is possible that participants swerved in and out of light sleep states during the scan. Since we did not identify significant differences regarding sleep-related complaints between OA-mTBI and OA-HCs as assessed with the HISC, we do not expect differences between groups related to this factor. However, we acknowledge that we did not verify if participants fell asleep as a limitation of this study. Third, although we do not have a reason to suspect that there would be differences between groups regarding caffeine consumption and/or its (temporary) effects on blood flow, we did not control for this factor. Lastly, due to the cross-sectional nature of this study, it is unknown how the identified alterations on brain connectivity and their interaction with PTCs evolve over time. Longitudinal studies are required to identify whether the observed effects on brain connectivity and the interaction between altered connectivity and severity of complaints can be replicated and predict the persistence of complaints. This knowledge might help defining more effective rehabilitation strategies for older adults at risk of developing persistent PTCs.

## Conclusions

Our findings on altered brain connectivity in OA-mTBI converged in visual(-cerebellar) and cognitive-language networks, some of which were associated with severity of complaints. These findings bring indirect evidence of a possible association of abnormal activation in brain networks with oculo-vestibular and cognitive impairments and warrant further investigation.

## Supplementary Information

Below is the link to the electronic supplementary material.

Supplementary file1 (DOCX 10.8 MB)

## Data Availability

Not applicable.
