# Supplementary material for: Effects of Mild Traumatic Brain Injury on Resting State Brain Network Connectivity in Older Adults
Source: Brain Imaging Behav. 2022 Apr 8;16(4):1863–72. doi: 10.1007/s11682-022-00662-5 (PMC9279274; doi:10.1007/s11682-022-00662-5)
Supplement: Supplementary file 1 — Supplementary file1 (DOCX 10.8 MB) [file 11682_2022_662_MOESM1_ESM.docx]

Appendix

1. **Supplementary Methods**

Our methodological approach for Group Independent Component Analysis (Group ICA) and multivariate statistical analysis was based on a previous study of our group that aimed for discriminating effects of mTBI and age in a group of adults with a broader age range (Bittencourt-Villalpando et al., 2021). This approach is described here again (using quotation marks) for readability purposes. Parameters that were adjusted for the present study are described outside of quotation marks.

- 1. ***Group Independent Component Analysis***

“The Group ICA fMRI Toolbox (GIFT^[[1]](#footnote-1)^; version 4.0b) was used to perform group-level spatial independent component analysis (Calhoun et al., 2001). The preprocessed fMRI data from” 45 participants “were decomposed into independent components (ICs) using group-information guided independent component analysis (GIG-ICA) (Du and Fan, 2013). The number of ICs (*N_c_*) was estimated using the minimum description length (MDL) criterion (Li et al., 2007). Subject-specific data reduction using two-step principal component analysis (PCA) first reduced the data to” 100 principal components “followed by group data reduction retaining *N_c_* ICs. The Infomax ICA algorithm (Bell and Sejnowski, 1995) was repeated 20 times using the built-in ICASSO^[[2]](#footnote-2)^ tool, which is used to estimate the stability and quality of the ICs. Additional parameters for ICASSO were bootstrapping with randomized initial condition, minimum cluster size of 16 components (0.8*20 ICA repetitions) and maximum cluster size of 20 components (same value as the number of ICA repetitions) (Himberg et al., 2004). The quality of components was quantified as the quality index (*I_q_*; range 0 to 1) and the minimum *I_q_* for inclusion of the component in the analysis was set at 0.9. The *N_c_* ICs from the best performing run were used as templates for subject-specific back-reconstruction using GIG-ICA (Du et al., 2014; Du and Fan, 2013). We identified the ICs considered to be ICNs, as opposed to physiological artefacts, by visually inspecting their aggregate spatial maps and their average power spectra. The identification of ICNs was done by authors MBV and HJvdH, independently. In accordance with previously published literature, the classification of components as ICNs was based on the following criteria: a) exhibiting peak activations primarily in gray matter, b) low spatial overlap with known vascular, ventricular motion and susceptibility artefacts and c) time courses dominated by low frequency fluctuations (Cordes et al., 2000). Differences were discussed until consensus was reached” (Bittencourt-Villalpando et al., 2021).

- 1. ***Connectivity assessment***

“All measures of connectivity were calculated using the MANCOVAN toolbox^[[3]](#footnote-3)^ (Allen et al., 2011) that is implemented in GIFT.

FNC was estimated as the Pearson’s correlation of pairs of TCs (Allen et al., 2011; Jafri et al., 2008). Subject-specific TCs were detrended and despiked using 3dDespike^[[4]](#footnote-4)^, then filtered using a fifth-order Butterworth low-pass filter with a high-frequency cut-off of 0.15 Hz. Subsequently, the variance associated with the motion parameter covariates was regressed out. For FNC statistical analysis, correlations were transformed to z-scores using Fisher’s transformation: *z*=atanh(FNC), as implemented in the MANCOVAN toolbox (Allen et al., 2011).

The ICN SMs were thresholded based on voxelwise t-statistics to limit the statistical analysis to voxels with strong and consisted activation across participants, as explained in Allen et al., (2011). The threshold for voxel selection per SM was set at $t>\mu+4\sigma$ across t-values (in the respective SM). GIG-ICA automatically generates Z-scored ICs (Du and Fan, 2013)” (Bittencourt-Villalpando et al., 2021).

- 1. ***Post-hoc Analysis***

Two week HISC-sev data from the UPFRONT study were used for determining complaint domains (van der Naalt et al. 2017). Only patients without missing complaint values were selected, resulting in a group of 541 patients. Subsequently, a matrix containing polychoric pairwise correlations was made for the entire group using the “polychoric_proc_missing” function (created by Luis Eduardo Garrido, 2017) in Matlab. This complaint × complaint matrix was then fed into the “community_louvain function” of the brain connectivity toolbox implemented in Matlab to find the optimal modular organization, i.e., subdividing the matrix into groups of elements (complaint domains) with maximized number of edges within and minimized number of edges between the groups (Blondel et al. 2008; Rubinov and Sporns 2010).

Subsequently, the HISC-sev scores of every participant in the current fMRI study were averaged across complaints within each domain. We used Spearman correlation coefficients (rho) to determine the association between complaint domains and altered brain connectivity using the “corr” function in Matlab.

All post-hoc tests were corrected for multiple comparisons at a significance level of α=0.05 using false discovery rate correction (FDR; Genovese et al. 2002).

1. **Supplementary Results**

An overview of the prevalence of post-traumatic symptoms within the older adults with mild traumatic brain injury (OA-mTBI) group can be found in Figs. S1 below. Post-traumatic symptoms are defined as complaints that were new or had their severity increased after mTBI.

- 1. ***Complaints and post-traumatic symptoms***


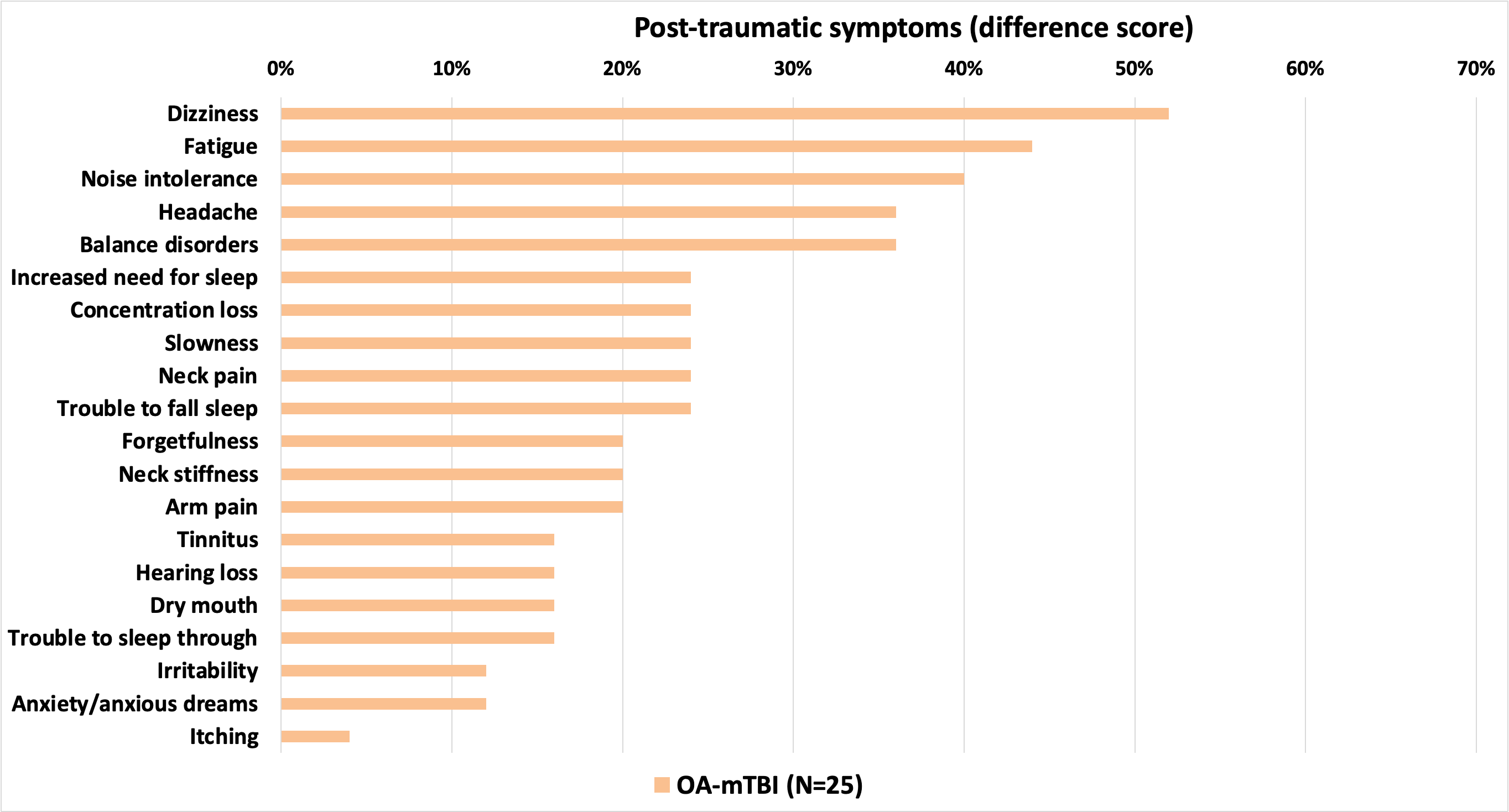


Figure S1 – Overview of the prevalence of post-traumatic symptoms (i.e. complaints that were new or had their severity increased after mTBI; %) for older adults with mTBI (OA-mTBI). Post-traumatic symptoms ordered by prevalence in the OA-mTBI group from highest to lowest.

- 1. ***Group ICA and RSNs***

The group ICA resulted in Nc = 15 ICs, which were all identified as ICNs (C = 15) and grouped into five functional domains (see Fig. S2). The final set included two ICNs for the Default-Mode (DM), five for the cognitive-control (Cog-C), three for the visual(-cerebellar) (Vis-CB), two for the sensorimotor (SMO), and three for a mixed domain. The mixed domain consists of three ICNs with high activation in more than one functional domain, namely ICN13 (cognitive-control and language domains; Cog-C/Lan), ICN 14 (sensorimotor, language and limbic domains; SMO/Lan/Lim), and ICN15 (auditory and cognitive-control domains; Aud/Cog-C).


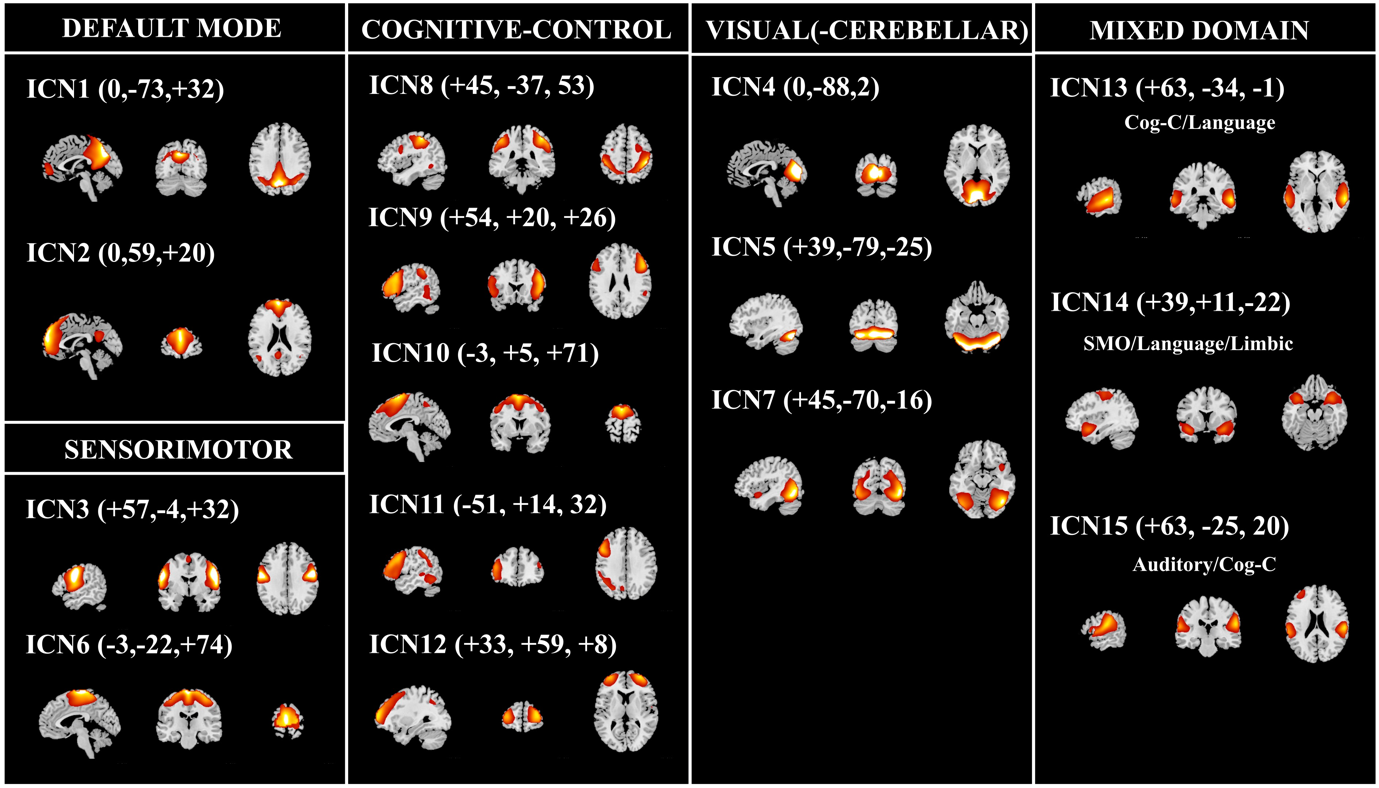


Figure S2 - Spatial maps of the 15 intrinsic connectivity networks (ICNs) identified as belonging to functional domains, thresholded at z-score > 3. Cog-C: cognitive-control; SMO: Sensorimotor.

- 1. ***Multivariate Analysis***

The results from the multivariate tests representing the significance of the covariates of interest in predicting SMI and FNC for the 15 identified ICNs are shown in the Fig. S3. Group, sqrt(HISC) and the interaction term sqrt(HISC) × group were retained as predictors for SMI of several ICNs. Age was retained as a predictor only for ICN12. None of the covariates of interest were retained as predictors for FNC.

FD was found to be predictor for SMI of only for ICN4, suggesting low motion contamination of ICN spatial maps. However, FD was found as a predictor for FNC, supporting the incorporation of FD as a nuisance covariate for the analysis of connectivity measures.


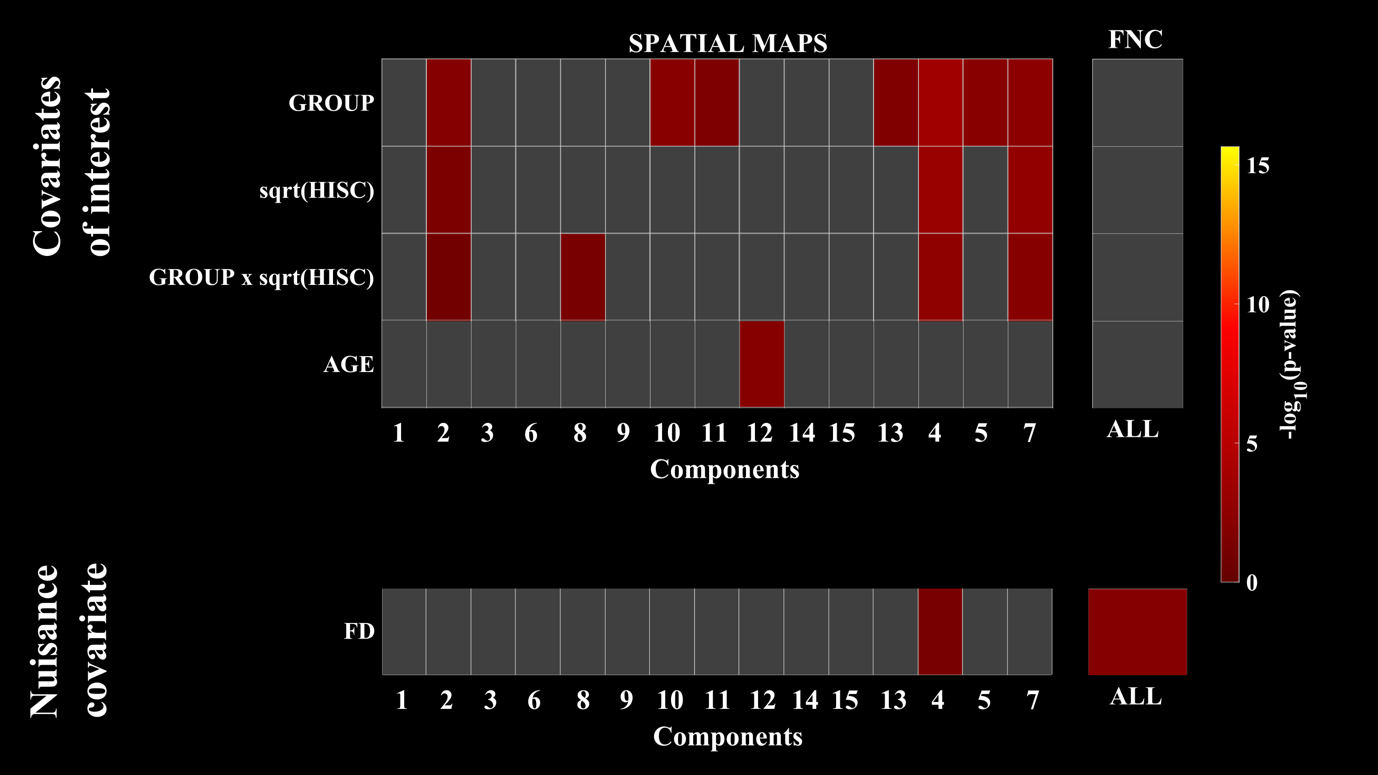


Figure S3 – Results from the multivariate tests showing the significance of the covariates of interest and nuisance covariates for spatial maps intensities, time courses spectra and FNC. Gray squares represent model terms that were not retained in the backward selection process (α = 0.05).

- 1. ***Post-hoc Analysis***

The resulting optimal community structure of the complaint × complaint polychoric correlations matrix consisted of five groups of elements, which were defined as Vestibular (DOM1), Auditory (DOM2), Cognitive-Fatigue ( Cog-Fatigue; DOM3), Physical (DOM4) and Anxiety-Sleep (DOM5) domains (see Fig. S4).
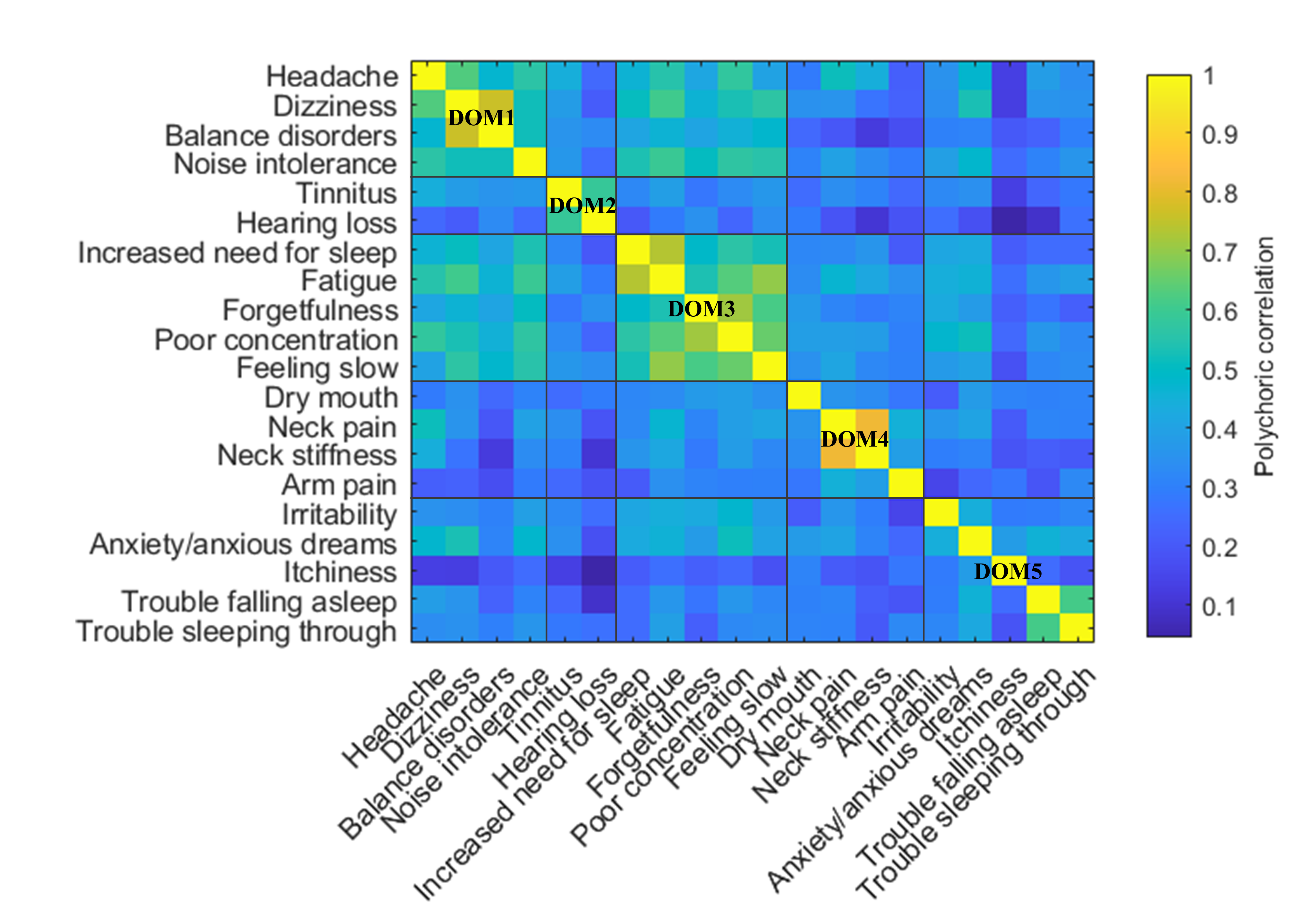
**Figure S4** – Optimal community structure of the complaint × complaint polychoric correlations matrix indicating five complaint domains

The association between complaint domains and altered brain connectivity for each of the main effects and interaction effects that were identified in the main statistical analysis is shown in Table S1 and Table S2.

Table S1 - Post-hoc analysis: association between main effects and complaint domains

| Main effect | Brain region | Cluster size (mm^3^) | Complaint Domain | rho | p-value |
| --- | --- | --- | --- | --- | --- |
| ICN13  (Cog-C/Lan) | lMTG | 378 | DOM1,  Vestibular | 0.55 | <0.001 |
|  |  |  | DOM3,  Cognitive-fatigue | 0.40 | 0.007 |
| ICN5  (Vis-CB) | rFG | 108 | - | - | - |

Cog-C/Lan: Cognitive-Control/Language; DOM: complaint domain; ICN: Intrinsic connectivity network lMTG: left middle temporal gyrus; rFG: right fusiform gyrus; Vis-CB: Visual-Cerebellar

Table S2 - Post-hoc analysis: association between interaction effects and complaint domains

|  |  |  |  | **OA-HC** | | **OA-mTBI** | |
| --- | --- | --- | --- | --- | --- | --- | --- |
| Interaction effect | Brain region | Cluster size (mm^3^) | Complaint Domain | rho | p-value | rho | p-value |
| ICN7  (Vis-CB) | aFG, mOG | 1512 | DOM1, Vestibular | - | - | 0.86 | <0.001 |
|  |  |  | DOM3, Cognitive-fatigue | - | - | 0.63 | 0.001 |
| ICN7  (Vis-CB) | Cerebellum VI and Crus I | 594 | DOM1, Vestibular | 0.65 | 0.002 | -0.58 | 0.002 |
|  |  |  | DOM2, Auditory | 0.63 | 0.003 | - | - |
| ICN4  (Vis-CB) | Cuneus | 324 | DOM1, Vestibular | - | - | 0.63 | 0.001 |
|  |  |  | DOM5, Anxiety-Sleep | - | - | 0.56 | 0.004 |

aFG: anterior fusiform gyri; DOM: complaint domain; ICN: intrinsic connectivity network; mOG: middle occipital gyri; OA-HC: older adult healthy control; OA-mTBI: older adult with mild traumatic injury; Vis-CB: Visual-Cerebellar

1. <http://mialab.mrn.org> [↑](#footnote-ref-1)
2. <http://www.cis.hut.fi/projects/ica/icasso> [↑](#footnote-ref-2)
3. http://www.mathworks.com/matlabcentral/fileexchange/27014-mancovan [↑](#footnote-ref-3)
4. http://afni.nimh.nih.gov/afni [↑](#footnote-ref-4)
